# The British Home for Incurables, Clapham

**Published:** 1893-06-03

**Authors:** 


### THE BRITISH HOME FOR INCURABLES, CLAPHAM.

A festival dinner in aid of the funds of this charity was held last week at the Hotel Metropole, Mr. C. E. Tritton, M.P., presiding. Amongst those present were the Earl and Countess Amherst, Lord Wick Browne, the Hon. Madeline Parnell, and Lieut.-General Sir F. and Lady Norman. The Chairman, in proposing the toast of the evening, said the institution was started 32 years ago, and admission was only granted to those who could be rightly designated "incurable." The objects of the charity were twofold—to provide a home for life for incurables, and pensions for life to the extent of £20 per annum to out-pensioners in connection with the Home. It was matter for congratulation that in a time of considerable depression the funds had been kept up fairly satisfactorily. The lease of the premises at Clapham had now expired, and the new home of the institution was to be at Norwood. The Chairman hoped that when the time came the new building might be opened by some member of the Royal family. The Princess May, to whom he wished every happiness in her approaching marriage, was a sincere friend to the Home. Funds were earnestly appealed for, and subscriptions and donations amounting to £4,900 were announced, this sum including £1,500 for a chapel for the new institution at Norwood from Miss Leicester, and £500 from Mr. Pinder Simpson.

